# Adjuvant treatment for triple negative breast cancer with residual tumor after neo-adjuvant chemotherapy. A single institutional retrospective analysis

**DOI:** 10.1016/j.breast.2021.08.004

**Published:** 2021-08-11

**Authors:** Nadia Bianco, Antonella Palazzo, Eleonora Pagan, Vincenzo Bagnardi, Monica Milano, Ana Paula De Maio, Marco Colleoni

**Affiliations:** a Division of Medical Senology, IEO, European Institute of Oncology, IRCCS, Milan, Italy; b Department of Statistics and Quantitative Methods, University of Milan-Bicocca, Milan, Italy; c Comprehensive Cancer Center, Medical Oncology Unit, Fondazione Policlinico Universitario A. Gemelli IRCCS, Rome, Italy

## Abstract

**Purpose:**

Incomplete response to neoadjuvant chemotherapy (NACT) in triple negative breast cancer (TNBC) patients is correlated to high risk of relapse. This study aimed to evaluate the role of adjuvant chemotherapy in TNBC with residual tumor after NACT.

**Methods:**

We retrospectively reviewed the outcome of patients with TNBC with residual tumor at surgery after a neoadjuvant treatment, followed by either adjuvant chemotherapy or observation. Primary endpoints were Disease Free Survival (DFS) and Overall Survival (OS).

**Results:**

Between January 2000 and December 2016, 223 patients with early TNBC operated at the European Institute of Oncology were eligible. A total of 83.4 % of patients received adjuvant chemotherapy after surgery. 90 patients received standard dose infusional regimens, while 96 patients (51.6 %) received oral metronomic chemotherapy. Adjusting the analysis by surgical stage and Ki67 value there was a benefit for DFS and OS in favor of the group that received postoperative chemotherapy (DFS-HR 0.58 p = 0.04; OS-HR 0.54, p = 0.02). At a subgroup analysis according to the different adjuvant treatments received, a benefit for metronomic chemotherapy versus no chemotherapy both for DFS (HR 0.46, p = 0.008) and OS (HR 0.45, p = 0.009) was reported.

**Conclusion:**

Our retrospective analysis in a large cohort of TNBC patients with residual disease after NACT confirms the benefit of adding a postoperative treatment to reduce risk of relapse and death. Based on these results, we suggest that the adjuvant therapy based on metronomic cyclophosphamide and methotrexate deserves further investigation in this patients population.

## Highlights

• Triple negative breast cancer is associated with worse prognosis and high rate of relapse.
• Different treatment strategies have been evaluated to manage TNBC disease in any stage.
• Pathological residual disease after a neoadjuvant chemotherapy represents an important prognostic factor.
• Adjuvant treatment strategies in this setting have shown an impact on outcome.
• Metronomic therapy could be a valid and interesting approach in this context.

## 1 Introduction

Despite the multimodal treatment, which includes surgery and chemotherapy, triple negative breast cancer (TNBC) is associated to a poor prognosis [1]. Currently, the standard treatment for TNBC patients is the neoadjuvant chemotherapy (NACT) which has been shown to be effective in reducing the size of locally advanced breast cancer and keeping the disease under control in the most aggressive subtypes.

Extensive efforts have been made to increase the pathological complete response (pCR) rate to NACT including chemo-dose intensification, by adding platinum compounds [2,3] and, more recently, with the use of immune checkpoint inhibitors [4], however about 40 % of TNBC patients did not achieve a pCR and experience tumor recurrence thereafter [5].

The use of non–cross-resistant chemotherapy drugs in the adjuvant setting might overcome the drug-resistant tumor clones resulting from NACT. In 2017 there was the evidence for a better disease-free survival (DFS) and overall survival (OS) with adjuvant capecitabine in a single randomized clinical trial for the subgroup of TNBC patients with pathological residual disease after NACT [6]. Before 2017, the use of post-neoadjuvant chemotherapy was not clearly supported by any scientific evidence. However the role of capecitabine in adjuvant setting for TNBC remains controversial [7].

Alkylating agents also showed high efficacy in TNBC. A retrospective analysis conducted on patients enrolled in two randomized clinical trials with node negative breast cancer, showed the benefits obtained from adjuvant classical CMF (cyclophosphamide/methotrexate/5-fluorouracil) in triple negative subtype [8]. Adjuvant chemotherapy with low dose and continuous schedule (metronomic) of cyclophosphamide and methotrexate (mCM) was investigated for prolonged adjuvant treatment in TNBC after standard adjuvant chemotherapy with a favoring benefit in node positive patients [9]. Thus, alkylating agents-based regimens could be considered as post-neoadjuvant chemotherapy for TNBC with residual disease. The aim of this retrospective study was to explore the benefit of adjuvant chemotherapy in a large series of TNBC patients with pathological residual disease after NACT. A comparison for benefit among different adjuvant regimens used was also investigated.

## 2 Material and methods

We retrospectively analyzed all consecutive patients with triple negative early breast cancer treated with a neoadjuvant chemotherapy, who underwent surgery at the European Institute of Oncology (EIO) Italy, with a pathologically invasive residual disease post NACT, collected from our institutional database between 2000 and 2016.

### 2.1 Patients

All patients evaluated in this analysis were treated with anthracycline with or without taxane, or anthracycline/taxane plus platinum compounds as neoadjuvant chemotherapy.

We considered all patients with a diagnosis of triple negative pathologically residual invasive breast cancer in the surgical specimen of the breast or axillary lymph nodes after completion of neoadjuvant chemotherapy. Triple negative breast cancer was defined as estrogen and progesterone receptors staining by immunohistochemistry (IHC) inferior to 1 % and human epidermal growth factor receptor 2 (HER2) negative with an IHC result of 0 or 1+ for cellular membrane protein expression or an in situ hybridization (ISH) negative result in accordance with recent reports [10,11].

A dedicated multidisciplinary team of breast cancer specialists discussed the post-operative (adjuvant) approach according to residual tumor at the final pathological report. All patients included in the analysis had a post-operative radiological staging to exclude the presence of early recurrence or distant metastases.

The following parameters were investigated for their prognostic role: clinical stage before NACT, pathological stage after NACT (defined as invasive residual tumor in breast or lymph nodes ypT0 or ypN0) according to TNM staging, lymph nodes metastasis (positive or negative nodes at the time of surgery), histological tumor size in residual invasive tumor (ypT1, ypT2, ypT3, ypT4), proliferation index (Ki67), peritumoral vascular invasion, adjuvant radiotherapy, postoperative approach (adjuvant chemotherapy or follow-up) and type of adjuvant regimen used.

We classified the adjuvant regimens in two groups based on their different schedule and dosage: one based on standard dose infusional regimens CMF/FU [i.e., classical CMF schema, intravenous CMF, infusional fluorouracil (FU)]**,** and the other based on oral metronomic chemotherapy (i.e., metronomic cyclophosphamide and capecitabine, mCC, or cyclophosphamide and methotrexate, mCM).

### 2.2 Statistical analysis

The primary study endpoint was to evaluate DFS and OS in women with early stage TNBC, who received NACT and who did not achieve pCR, with attention on the role of the postoperative chemotherapy.

DFS was defined as the time from surgery to events such as relapse (including ipsilateral breast recurrence, invasive or in situ), appearance of a second primary cancer (including contralateral breast cancer, invasive or in situ), or death, whichever occurred first. OS was defined as the time from surgery until the date of death (from any cause).

Active follow-up was conducted to determine patient status as of March 2019.

The DFS and OS functions were estimated using the Kaplan–Meier method. Cox proportional hazards univariable and multivariable models were used to estimate the DFS and OS hazard ratios (HR) for adjuvant CT. Variables significantly associated (p < 0.05) with adjuvant CT were retained in the multivariable models.

Patients' and tumor’ characteristics were reported with absoulte and relative frequencies in case of categorical variables, and with median and interquartile range (IQR) in case of continuous variables. Categorical variables and treatment groups were compared with Fisher's exact test while the Wilcoxon rank-sum test was used to compare medians of Ki67 between treatment groups.

All analyses were carried out with the SAS software v. 9.4 (SAS Institute, Cary, NC).

## 3 Results

Between January 2000 and December 2016, at EIO fulfilled the inclusion criteria of the analysis.

The baseline characteristics of patients are shown in Table 1 and Table 2.

**Table 1:** Patients’ characteristics (N = 223 enrolled patients).

	Adjuvant chemotherapy		*P*-value
	No (N = 37)	Yes (N = 186)	
Type of adjuvant chemotherapy			
No chemotherapy	37	0	
CMF/FU∗	0	89	
mCM/mCC	0	97	
**Year of surgery**			0.05
Before 2003	8 (30.8)	18 (69.2)	
2003–2006	11 (22.9)	37 (77.1)	
2007–2010	6 (9.4)	58 (90.6)	
After 2010	12 (14.1)	73 (85.9)	
**Age (year)**			0.69
≤35	5 (11.9)	37 (88.1)	
36–64	29 (17.9)	133 (82.1)	
≥65	3 (15.8)	16 (84.2)	
**Stage at neoadjuvant chemotherapy**			0.72
II	19 (15.8)	101 (84.2)	
III	18 (18.0)	82 (82.0)	
Unknown	0	3 (100.0)	
**Type of neoadjuvant chemotherapy**			0.40
Anthracycline	7 (22.6)	24 (77.4)	
Anthracycline + Taxane	15 (17.2)	72 (82.8)	
Anthracycline/Taxane + Cisplatin	13 (13.3)	85 (86.7)	
Other	2 (28.6)	5 (71.4)	
**Stage at surgery**			0.001
I	21 (26.3)	59 (73.8)	
II	6 (6.5)	86 (93.5)	
III	10 (19.6)	41 (80.4)	
**Histology**			0.54
Ductal	35 (17.3)	167 (82.7)	
Other	2 (9.5)	19 (90.5)	
**ypT**			0.07
ypT0/is/X	1 (7.1)	13 (92.9)	
ypT1	24 (22.2)	84 (77.8)	
ypT2	6 (9.1)	60 (90.9)	
ypT3	4 (13.3)	26 (86.7)	
ypT4	2 (40.0)	3 (60.0)	
**ypN**			0.37
Negative	23 (18.7)	100 (81.3)	
Positive	14 (14.0)	86 (86.0)	
**M**			1
X	1 (16.7)	5 (83.3)	
0	36 (16.6)	181 (83.4)	
**Peritumoral vascular invasion**			1
No	27 (16.9)	133 (83.1)	
Yes	10 (16.1)	52 (83.9)	
Unknown	0	1 (100.0)	
**Radiotherapy**			0.35
No	9 (22.5)	31 (77.5)	
Yes	28 (15.4)	154 (84.6)	
Unknown	0	1 (100.0)	
**Ki67 (%)**			0.04
<20 %	12 (28.6)	30 (71.4)	
≥20 %	25 (13.8)	156 (86.2)	
**Ki67 (%), median (IQR)**	40.0 (15.0–65.0)	60.0 (28.0–80.0)	0.07

∗1 patient received FU.

**Table 2:** Patients’ characteristics according to type of adjuvant treatments.

	Adjuvant chemotherapy			*P*-value II vs I	*P*-value III vs I	*P*-value III vs II
	No [I] (N = 37)	CMF/FU [II] (N = 89)	mCM/mCC [III] (N = 97)			
**Year of surgery**				0.06	<0.001	<0.001
Before 2003	8 (30.8)	17 (65.4)	1 (3.8)			
2003–2006	11 (22.9)	10 (20.8)	27 (56.3)			
2007–2010	6 (9.4)	16 (25.0)	42 (65.6)			
After 2010	12 (14.1)	46 (54.1)	27 (31.8)			
**Age (year)**				0.27	1	0.06
≤35	5 (11.9)	24 (57.1)	13 (31.0)			
36–64	29 (17.9)	59 (36.4)	74 (45.7)			
≥65	3 (15.8)	6 (31.6)	10 (52.6)			
**Stage at neoadjuvant chemotherapy**				0.70	0.85	0.66
II	19 (15.8)	49 (40.8)	52 (43.3)			
III	18 (18.0)	37 (37.0)	45 (45.0)			
Unknown	0	3 (100.0)	0			
**Type of neoadjuvant chemotherapy**				0.71	0.01	<0.001
Anthracycline	7 (22.6)	20 (64.5)	4 (12.9)			
Anthracycline + Taxane	15 (17.2)	40 (46.0)	32 (36.8)			
Anthracycline/Taxane + Cisplatin	13 (13.3)	27 (27.6)	58 (59.2)			
Other	2 (28.6)	2 (28.6)	3 (42.9)			
**Stage at surgery**				<0.001	0.02	0.07
I	21 (26.3)	21 (26.3)	38 (47.5)			
II	6 (6.5)	47 (51.1)	39 (42.4)			
III	10 (19.6)	21 (41.2)	20 (39.2)			
**Histology**				1	0.35	0.34
Ductal	35 (17.3)	82 (40.6)	85 (42.1)			
Other	2 (9.5)	7 (33.3)	12 (57.1)			
**ypT**				0.05	0.18	0.31
ypT0/is/X	1 (7.1)	4 (28.6)	9 (64.3)			
ypT1	24 (22.2)	36 (33.3)	48 (44.4)			
ypT2	6 (9.1)	34 (51.5)	26 (39.4)			
ypT3	4 (13.3)	13 (43.3)	13 (43.3)			
ypT4	2 (40.0)	2 (40.0)	1 (20.0)			
**ypN**				0.25	0.70	0.46
Negative	23 (18.7)	45 (36.6)	55 (44.7)			
Positive	14 (14.0)	44 (44.0)	42 (42.0)			
**M**				1	1	1
X	1 (16.7)	2 (33.3)	3 (50.0)			
0	36 (16.6)	87 (40.1)	94 (43.3)			
**Peritumoral vascular invasion**				0.83	1	0.74
No	27 (16.9)	62 (38.8)	71 (44.4)			
Yes	10 (16.1)	26 (41.9)	26 (41.9)			
Unknown	0	1 (100.0)	0			
**Radiotherapy**				0.64	0.19	0.24
No	9 (22.5)	18 (45.0)	13 (32.5)			
Yes	28 (15.4)	70 (38.5)	84 (46.2)			
Unknown	0	1 (100.0)	0			
**Ki67 (%)**				0.01	0.17	0.23
<20 %	12 (28.6)	11 (26.2)	19 (45.2)			
≥20 %	25 (13.8)	78 (43.1)	78 (43.1)			
**Ki67 (%), median (IQR)**	40.0 (15.0–65.0)	70.0 (35.0–85.0)	40.0 (23.0–70.0)	0.003	0.66	<0.001

Patients had clinical stage II or III at diagnosis. A downstaging after neoadjuvant chemotherapy at stage I was obtained in 36 % patients (80/223) while 92 of 223 (41.2 %) had a pathological residual disease of stage II and 51 (22.9 %) of stage III. One hundred eighty-one patients (81.2 %) had pathological residual disease with high proliferative index (Ki67 above 20 %). Sixty-two of 223 (27.8 %) had vascular invasion evidence in pathological residual disease.

One hundred eighty-six out of 223 patients (83.4 %) received an adjuvant chemotherapy after surgery: 89 patients (39.9 %) received standard infusional regimens (CMF/FU) while 97 patients (43.4 %) received metronomic regimens (mCM/mCC). Thirty-seven patients (16.6 %) did not receive further adjuvant chemotherapy. We found only one patient receiving FU and one patient receiving mCC.

Sixteen percent of stage II and 18 % of stage III patients did not received adjuvant chemotherapy after surgery.

The majority of patients received adjuvant radiotherapy (81.6 %). Among the 28 patients treated with radiotherapy and who did not receive adjuvant chemotherapy, 12 received complementary radiotherapy while 16 a loco-regional one. Of 154 patients treated with radiotherapy who received adjuvant chemotherapy: 74 received complementary radiotherapy (29 in patients treated with CMF/FU, 45 in patients treated with mCM/mCC) and 79 received loco-regional radiotherapy (40 in patients treated with CMF/FU, 39 in patients treated with mCM/mCC). For 1 patient treated with CMF the detail on radiotherapy was missing.

All patients’ characteristics evaluated were well balanced between groups, comparing patients who received adjuvant chemotherapy and did not (yes versus no) and also among different type of adjuvant treatment groups (no versus CMF/FU versus mCM/mCC), except for stage and Ki67 (p = 0.01 and p = 0.04, respectively).

The median follow-up time was 9.9 years (IQR 4.1–12.6 years).

Overall, 105 DFS events, 58 of which were distant metastasis, and 88 OS events were observed (Table 3).

**Table 3:** Survival outcomes according to type of adjuvant treatments.

	Adjuvant chemotherapy			
	No (N = 37)	Yes		
		Overall (N = 186)	CMF/FU (N = 89)	mCM/mCC (N = 97)
**Disease-free survival (DFS)**				
Observed events, N (%)	19 (51.3)	86 (46.2)	50 (56.2)	36 (37.1)
Loco-regional events, N	4	30	15	15
Distant metastases, N	13	45	28	17
Other events, N	2	11	7	4
5-yr DFS (95 % CI)	52.5 (33.6–68.3)	55.9 (48.0–63.0)	45.9 (34.4–56.7)	64.5 (53.7–73.3)
10-yr DFS (95 % CI)	47.2 (28.0–64.3)	47.8 (39.3–55.8)	33.6 (21.6–46.0)	59.8 (48.5–69.4)
HR (95 % CI) [crude]	Ref.	0.80 (0.49–1.32)	1.09 (0.64–1.86)	0.59 (0.34–1.02)
HR (95 % CI) [adjusted by stage and Ki67]	Ref.	0.58 (0.35–0.97)	0.74 (0.43–1.30)	0.46 (0.26–0.81)
p-value [adjusted by stage and Ki67]		0.04	0.30	0.008
**Overall survival (OS)**				
Observed deaths, N (%)	19 (51.3)	69 (37.1)	39 (43.8)	30 (30.9)
5-yr OS (95 % CI)	55.6 (37.0–70.7)	66.1 (58.4–72.8)	60.7 (48.9–70.5)	71.2 (60.6–79.4)
10-yr OS (95 % CI)	43.7 (25.6–60.5)	59.4 (51.1–66.8)	50.2 (37.3–61.7)	67.1 (56.1–76.0)
HR (95 % CI) [crude]	Ref.	0.69 (0.41–1.14)	0.88 (0.51–1.53)	0.53 (0.30–0.94)
HR (95 % CI) [adjusted by stage and Ki67]	Ref.	0.54 (0.32–0.91)	0.63 (0.36–1.12)	0.45 (0.25–0.81)
p-value [adjusted by stage and Ki67]		0.02	0.11	0.008

**Fig. 1:**
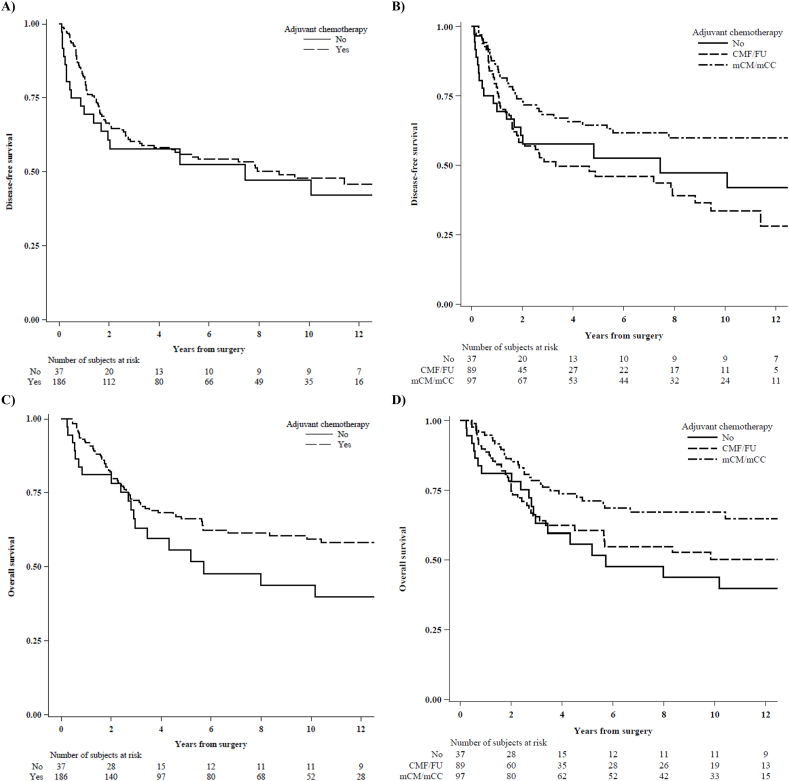
Disease-free survival (panel A and B) and overall survival (panel C and D) according to adjuvant treatments.

Fig. 1 reported DFS and OS curves according to adjuvant treatment.

Adjusting the analysis by stage at surgery and Ki67 value, we found a difference in DFS between patients that received adjuvant chemotherapy versus those that did not receive further chemotherapy (adjusted HR 0.58; 95 % CI: 0.35–0.97; p-value = 0.04; Table 3).

Comparing the two adjuvant treatment groups, we found no difference in DFS between the group that received infusional chemotherapy CMF/FU versus no chemotherapy (adjusted HR 0.74; 95 % CI: 0.43–1.30; p-value = 0.30) and a benefit from mCM/mCC versus no chemotherapy (adjusted HR 0.46; 95 % CI: 0.26–0.81; p-value = 0.008; Table 3).

In term of OS, adjusting for stage and Ki67, we also found a difference in favor of group that received chemotherapy (adjusted HR 0.54; 95 % CI: 0.32–0.91; p-value = 0.02; Table 3). The same OS advantage was found comparing the group mCM/mCC based chemotherapy versus no chemotherapy (adjusted HR 0.45; 95 % CI: 0.25–0.81; p-value = 0.008; Table 3).

## 4 Discussion

Triple negative breast cancer patients who do not achieve a pathological complete response (pCR) have a higher risk of relapse and death (76 % and 84 % respectively) when compared with patients who obtained a pCR (pCR Event free survival HR 0.24; 95 % CI 0.18–0.33; pCR OS HR 0.16; 95%CI 0.11–0.25) [5]. Many efforts have been done to evaluate if adding a non-crossing postoperative agent could improve outcome of these patients at higher risk of relapse.

Our retrospective analysis on 223 patients with a median time of observation of 10 years confirms that adding adjuvant treatment to a residual triple negative disease after NACT reduce of about 40 % the risk of relapse (HR 0.58; 95 % CI: 0.35–0.97; p-value = 0.04) and of about 50 % the risk of death (HR 0.54; 95 % CI: 0.32–0.91; p-value = 0.02).

Up to date only one randomized phase III trial on Asian patients with residual invasive tumors or lymph-node metastasis after optimal NACT investigates the use of capecitabine at a dose of 1250 mg per square meter of body-surface area, twice per day, on days 1–14 every 3 weeks for six or eight cycles versus placebo. In this trial capecitabine prolonged disease-free survival and overall survival, with a particular benefit in triple negative subtype (DFS HR: 0.58; 95 % CI: 0.39–0.87; OS HR: 0.52; 95 % CI: 0.30–0.90) [6]. However, it should be noted that this trial was designed for HER2 negative breast cancer, of which triple negative tumors were 30 % of overall study population (N = 286) and limited benefit was demonstrated for patients with hormone receptor positive. Although, even if more data are necessary in non-Asian patients, according to breast cancer guidelines, postoperative capecitabine at standard dose and schedule may be offered to patients who do not achieve a pCR after optimal NACT [12,13].

Considering more recent data the use of adjuvant capecitabine remains controversial. In a larger randomized phase III trial for operable triple negative disease who received standard (neo)adjuvant chemotherapy and including about 80 % of high-risk patients with stage II-III, adding capecitabine at 1000 mg/m2 orally two times per day on days 1–14 every 3 weeks failed to improve DFS (adjusted HR according to stratification factors, 0.79; 95 % CI, 0.61 to 1.03; P = 0.082) [14]. In this study were also included patients with triple negative residual disease after NACT who received capecitabine as postoperative treatment (N = 67).

Our retrospective analysis includes a relevant number of triple negative breast cancer patients (N = 223) mainly treated with standard neoadjuvant anthracycline and taxane regimens, other than combination including platinum salts. The majority of our patients received a postoperative treatment, including 39.9 % with infusional chemotherapy and 43.5 % with metronomic oral chemotherapy. Only a minority of patients did not receive any type of postoperative treatment (16.6 %).

Early studies [15] suggest that regimens including antimetabolites and alkylating agents such as cyclophosphamide may be effective in triple-negative tumors. In the preoperative setting there is an evidence of higher pathological complete response rate with high-dose alkylating agents in triple negative breast cancer with p53 mutation [16]. Moreover, in a metanalysis comparing anthracycline-based adjuvant regimens with non – anthracycline based (including CMF like regimen) in women with early-stage breast cancer was provided evidence of an interaction between HER2 status and responsiveness to adjuvant anthracyclines with no benefit from adjuvant anthracyclines regimens in patients with HER2-negative disease, raising the issue for a better selection of patients that could be benefit from non-anthracycline adjuvant regimens [17].

Analyzing outcome in our study population postoperative metronomic chemotherapy with cyclophosphamide and methotrexate was able to reduce of 54 % the risk of relapse (HR 0.46; 95 % CI: 0.26–0.81) and of 55 % the risk of death (HR 0.45; 95 % CI: 0.25–0.81).

The benefit showed with metronomic chemotherapy in our analysis is in line with the results of IBCSG 22–00 trial and SYSUCC 001 trial. The first trial was an open-label, two-arm, phase III, randomized study to evaluate efficacy and safety of the 12-month CM maintenance regimen versus no CM after standard adjuvant chemotherapy for ER and PgR negative (<10 %) with any HER2 status of early breast cancer. In this analysis metronomic CM showed a trend toward benefit observed in high-risk patient population with node-positive and triple negative subtype (N = 340) with an estimated 5-year DFS of 72.5 % for the CM maintenance group and was 64.6 % for the no-CM group (HR, 0.72; 95 % CI: 0.49 to 1.05) [9]. More recently the results of SYSUCC-001 trial indicates that the addition of low-dose capecitabine as maintenance therapy for 1 year following standard adjuvant treatment, compared with placebo, significantly improve disease-free survival in patients with early-stage triple negative breast cancer (HR for risk of recurrence or death, 0.64; 95 % CI: 0.42–0.95; P = 0.03). In this study the majority of patients received previous anthra-taxane based chemotherapy as adjuvant regimen and only a minority (3–5%) received chemotherapy as neoadjuvant/adjuvant setting [18]. These evidences and our larger analysis on benefit from prolonging adjuvant chemotherapy with metronomic schedule open an attractive approach for triple negative early breast cancer treatment. The magnitude of benefit derived from the low dose maintenance schedule could be explained by its mechanism of action including the DNA damage induced by continuous exposure that may significantly improve response rate reducing burden of chemotherapy related toxicity. Moreover the main targets of continuous low dose chemotherapeutics are also the endothelial cells of tumor blood vessels with effective tumor control and most recently among the new mechanisms identified for metronomic chemotherapy there is the restoration of the anticancer effect of the immune system [19].

In our analysis overall population had a very short prognosis. One possible explanation could be related to the old regimens of chemotherapy used in the neoadjuvant setting and also to the high percentage (64.1 %) of patients with a residual pathological stage of II and III after NACT.

About 43 % of patients who did not receive adjuvant chemotherapy were at pathological stage II-III.

Considering that there were no clear data on adjuvant chemotherapy in patients with residual disease after NACT at the time of our observation, the patients' selection and adjuvant treatment decision was based not only on pathological staging and biological features at the time of surgery, but also were evaluated the tolerance to NACT, general clinical condition and patient's preference. These factors could affect the overall prognosis in our population, in contrast with the more recent literature data, potentially confirming the benefit of an adjuvant treatment in all patients with a residual TNBC pathological disease.

The present analysis has several limitations including its retrospective nature and the small sample size within adjuvant treatment group. Other weakness is represented by the lack of information on some relevant prognostic factors as BRCA and TILs status after NACT.

In conclusion, our retrospective analysis on a large sample of triple negative breast cancer with residual disease after NACT confirm the benefit of postoperative treatment in reducing the risk of relapse and death. Although the analysis between the postoperative treatment group is exploratory, the benefit observed for adjuvant mCM according to recent literature data suggest further investigation with this regimen in this high-risk patient population.

## Funding

No external funding was used in the preparation of this article.

## Ethical approval

This article does not contain any studies with animals performed by any of the authors. All procedures performed in studies involving human participants were in accordance with the ethical standards of the institutional and/or national research committee and with the 1964 Helsinki declaration and its later amendments or comparable ethical standards.

## Collection data

Data collection has been performed under the normative regulations, indications and restrictions on the matter of retrospective clinical studies, according to the Italian deliberazione 01 Marzo 2012, Gazzetta Ufficiale n.72 del 26 Marzo 2012 and subsequent changes. All the patients signed the institutional informed consent for using their anonymised clinical data for scientific purposes.

The datasets generated during and/or analyzed during the current study are not publicly available due to institutional restrictions but are available from the corresponding author on reasonable request.

## Declaration of interests

The authors declared no conflicts of interest. Contributions All authors contributed equally to this paper. All authors made substantial contributions to conception and design, acquisition of data, analysis, and interpretation of data. All authors read and approved the final manuscript.
